# Evaluating the impact of delayed study startup on accrual in cancer studies

**DOI:** 10.1016/j.conctc.2025.101562

**Published:** 2025-10-23

**Authors:** Isuru Ratnayake, Anh-Tuan Do, Daniel Gajewski, Sam Pepper, Oluwatobiloba Ige, Natalie Streeter, Tara L. Lin, Matthew McGuirk, Byron Gajewski, Dinesh Pal Mudaranthakam

**Affiliations:** a Department of Biostatistics & Data Science, University of Kansas Medical Center, Kansas City, KS, USA; b The University of Kansas Cancer Center– Accelerated Cancer Education (ACE) Summer Intern 2023, Piper High School, Kansas City, KS, USA; c Department of Biostatistics & Data Science Summer Intern 2023, Rockhurst High School, Kansas City, MO, USA; d The University of Kansas Cancer Center, Kansas City, KS, USA

**Keywords:** Clinical trials, Study activation, Trial accrual, Study startup, Oncology studies

## Abstract

**Background:**

Drug development in cancer medicine relies on high-quality clinical trials, and the success of these trials depends on the design, optimization, and execution. Delays often arise from the study startup process, which can take 6 months or more. Complex challenges, including regulatory hurdles, contract negotiations, and inefficiencies in site activation, contribute to these delays. Streamlining these processes is critical to accelerating patients' access to potentially life-saving therapies.

**Method:**

Data from the University of Kansas Cancer Center (KUCC) were used to analyze studies initiated between 2018 and 2022. The accrual percentage was computed based on the number of enrolled participants and the desired accrual goal. Accrual success was determined by comparing the enrollment rate to predefined threshold values (50 %, 70 %, or 90 %).

**Results:**

Studies that achieve or surpass the 70 % accrual threshold typically exhibit a median activation time of 140.5 days. In contrast, studies that fall short of the accrual goal tend to have a median activation time of 187 days, indicating shorter median activation times are associated with successful studies. The Wilcoxon rank-sum test (W = 13,607, p = 0.001) indicated that early-phase studies had significantly longer activation times than late-phase studies. We also conducted the study with 50 % and 90 % accrual thresholds; our findings remained consistent.

**Conclusions:**

Longer activation times are associated with lower project success, and early-phase studies tend to be more successful than late-phase studies. Therefore, by reducing impediments to the approval process, we can facilitate quicker approvals, increasing the success of studies regardless of phase.

## 1 Introduction

The study startup process is a crucial step that includes regulatory, contractual, legal, and operational components in clinical trials, safeguarding both study quality and participant safety [1]. Common obstacles during study start-up include delays in reaching agreements on financial terms, insufficient sponsor-secured funding, design challenges whose suitability is confirmed only at the protocol review stage, and slow committee evaluations [2]. These problems are not unique to trials in the United States (U.S.); they also hinder studies worldwide. A recent report showed that study start-up and recruitment proceed more slowly in Europe than in the U.S. [3]. Studies show that the time required to activate a study is often inversely related to its enrollment rate, making lengthy start-up timelines a critical barrier to accrual success [4,5].

To understand the challenges that delay study start-up, we must first examine the start-up process itself. Launching a study requires close coordination among multidisciplinary teams to maintain a comprehensive perspective throughout the entire process. At academic medical centers, a unique component of this process is the institutional scientific review, in which study protocols are rigorously evaluated by Scientific Review Committees (SRCs). These committees serve as an initial filter, ensuring that only high-merit studies with strong scientific justification and operational feasibility proceed to the Institutional Review Board (IRB) for ethical review and protection of human subjects. Alongside committee review, centralized coverage analyses for multisite clinical trials have been shown to reduce risk for sites and patients, predict budget requirements, and help shorten trial startup times, which can hinder patient accrual and access to trials [6].

Once the SRC approves a study, the IRB then evaluates the protocol to ensure compliance with ethical and regulatory standards that protect human participants [1]. As part of the clinical trial approval process, the National Cancer Institute (NCI) requires that trials conducted at NCI-designated cancer centers undergo both institutional scientific review and IRB evaluation [7]. In contrast, clinical cancer research conducted at non-NCI-designated centers in the U.S. or at international institutions may not be subject to the scientific review requirement [8]. Additionally, depending on the institution or site, further internal reviews may also be conducted. Collectively, these serial reviews and approvals contribute to prolonged activation timelines [7].

The NCI's Operational-Efficiency Working Group sets an aspirational target of 90 days for the entire study start-up process. Yet, real-world experience is less encouraging: an Association of American Cancer Institutes (AACI) survey of 61 North American centers found a median start-up interval of 167 days [9]. The University of Kansas Cancer Center (KUCC), a NCI-designated Comprehensive Cancer Center, leads statewide efforts in cancer clinical trials across Kansas and western Missouri. KUCC has demonstrated that rigorous dashboard tracking and milestones can keep many protocols within its internal 90- and 120-day goals [1].

In response to the complex and often challenging steps involved in study startup, KUCC implemented a web-based platform—Trial Review and Approval for Execution (TRAX)—in August 2020 to systematically track key milestones, dates, and activities throughout the startup process. TRAX logs every cancer-related protocol and records a time stamp at each step of KUCC's sequential SRC pathway [1]. Reviews begin with the Disease Working Group (DWG), which assesses clinical need and strategic fit. They then proceed to the Executive Resourcing Committee (ERC), which evaluates operational feasibility and resource requirements. The process concludes with the Protocol Review and Monitoring Committee (PRMC). This independent body assesses scientific merit, statistical rigor, and ethics. The platform incorporates clear, committee-specific review guidelines, ensuring that reviewers apply the correct criteria at each stage, and it preserves the complete decision history as the study progresses. After SRC clearance, TRAX continues to track the protocol through IRB approval to final activation. This comprehensive tracking enhances transparency, streamlines handoffs, and provides actionable metrics that help KUCC reduce start-up timelines and quickly open appropriate trials to patients.

While TRAX tracks operational delays, enrollment remains the ultimate yardstick of trial success, and its pace is shaped by factors beyond workflow alone. In recent years, many anticancer agents have received regulatory approval based on clinical trials that met their enrollment targets and were completed on schedule [[10], [11], [12], [13]]. Yet trial start-up within academic centers remains slow and complex, and poor accrual is a recurring bottleneck. In contrast, previous studies have shown that accrual performance varies by study phase and funding source [14,15]; few have examined how these variables interact with activation timelines within an academic cancer center with a structured, internal scientific review process. Our study adds to the existing literature by leveraging five years of data from KUCC's Trial Review and Approval for Execution (TRAX) system—a centralized tracking platform—to quantify the relationship between startup duration, study characteristics, and accrual success. By doing so, we not only validate prior findings using more recent, operationally specific data but also highlight new, practical opportunities to improve study prioritization and workflow efficiency in the activation process.

Guided by these considerations, our primary aim was to examine whether study activation time is associated with accrual success, testing the hypothesis that more extended activation periods are associated with lower participant enrollment. We also discussed how activation times vary by study phase and funding source and assessed their potential associations with accrual outcomes. Establishing these relationships will illustrate how approval timelines correlate with enrollment success, providing sponsors and institutions with evidence to identify and address delays in the trial start-up process and to optimize future activations.

## 2 Methods

The dataset was extracted from the Clinical Trial Management System (CTMS), powered by WCG Velos. The platform is maintained by the Department of Biostatistics & Data Science at the University of Kansas Medical Center, which serves as an enterprise CTMS system used for both cancer and non-cancer studies. There is a specific module labelled as eCompliance that tracks the entire study start-up process based on the defined workflows for KUCC.

The dataset includes studies conducted by KUCC between January 1, 2018, and December 31, 2022, inclusive. During this period, 720 new studies entered the study startup process, and each was categorized as either closed or in the enrollment phase. A “closed” clinical trial refers to a study that has either completed participant accrual at all sites or has been terminated by the review committee. An “enrolling” study is still actively recruiting participants. Of the total studies reviewed, 519 (72 %) were closed, while 201 (28 %) were still enrolling at the time of data extraction. Among the 519 closed studies, 315 (60.7 % of 519) completed accruals across all sites, and 204 (39.3 % of 519) were terminated by the review committees without reaching accrual goals. For this analysis, our analytical data set included only the 315 studies that were closed with completed accruals across all sites. Terminated studies (n = 204) and enrolling studies (n = 201) were excluded. No additional studies were removed due to missing values in any of the study variables (Accrual Success, Activation Days, or Study Phase).

The **Accrual Success** variable reflects the success of the accrual process (Equation (1)). It is a dichotomous outcome variable (1 = success; 0 = fail), defined by whether the percentage of enrolled patients meets a predefined threshold level (*k*) after study activation. A study is categorized as “successful” if it achieves or surpasses a predetermined *k* threshold; values of k∈{0.5,0.7,0.9} which are equivalent to 50 %, 70 %, and 90 % respectively, were examined.

(1) $$Accrual\;Success=\left\{ \begin{array}{c} 0:\frac{number\;of\;enrollments}{desired\;accrual\;goal}<k\\ 1:\frac{number\;of\;enrollments}{desired\;accrual\;goal}\geq k \end{array}\right.\text{.}$$

Equation (1) formalizes our study's definition of accrual success and classifies each trial as meeting or falling short of the predefined enrollment benchmark. In this study, we excluded clinical trials from the analysis under several conditions: clinical trials that were still enrolling patients, and which had not yet met the threshold level (*k),* clinical trials with undefined Accrual Success; clinical trials missing values of phase (i.e., Phase=N/A); and clinical trials where the number of days from DWG approval to activation is unknown.

To examine the association between accrual success and the time required to activate a study, we defined **Activation Days** as the number of business days between DWG approval and the date the study is officially ready to begin enrolling participants. Any days on sponsor hold during this period have been deducted from the total Activation Days. Sponsor holds are typically excluded from study activation timeline metrics because they reflect external factors beyond the control of the site or the internal study teams [16]. These holds often arise from sponsor-regulatory, budgetary, or strategic decisions and do not reflect the operational efficiency of sites or other stakeholders. By excluding these periods, the timeline reflects only active progress phases, providing a more accurate and fair measure of study startup performance. Ultimately, this approach focuses on the parts of the process that can be improved or streamlined to support study success rather than on delays outside the trial team's control.

This variable can be expressed as follows:

(2) ActivationDays=A−B.

Here,

A=f(studyactivationdate−DWGapprovaldate),

B=f(sponsorholdenddate−sponsorholdstartdate).

The function f(.) in Equation (2) calculates the business days between any given calendar dates and returns a positive integer as the output; thus, this function excludes weekends and U.S. holidays. For example, if a clinical trial received its DWG approval on October 7, 2022, and was activated on May 12, 2023, with an 8-day sponsor hold, the Activation Days are equal to 152 business days. This calculation is explained as follows:

ActivationDays=f(May12,2023−October7,2022)−f(July5,2022−June23,2022)=152

It's important to note that not all studies have experienced sponsor holds.

Another essential objective of the study is to examine the association between the study phase (i.e., pilot, I, II, III, and IV) and the success of the accrual process. To investigate this, a dichotomized explanatory variable (early-phase, late-phase) is introduced and named **Study Phase**, which is described in Equation (3):

(3) $$Study\;Phase=\left\{ \begin{array}{c} 0=early-phase:Phase\in \left(pilot,I,I/II\right)\;\\ 1=late-phase:Phase\in \left(II,II/III,III,IV\right) \end{array}\right.\text{.}$$

To denote various funding categories, the **Study Source** variable was included. This is a nominal categorical variable with five categories: Externally Peer-Reviewed/Federal Funding, Industrial/Pharmaceutical, Internal Institutional/Investigator Initiated, External Institutional/Investigator Initiated, and National/Cooperative Group/Consortium.

We conducted a comprehensive statistical analysis using various techniques. Initially, we constructed box-and-whisker plots for the Accrual Success and Study Phase variables to assess the distribution of Activation Days visually. Given the non-normal distribution of the data, we applied the Wilcoxon rank-sum test to compare the median Activation Days between the Accrual Success and Study Phase groups. The Kruskal-Wallis test was employed to compare the median Activation Days across different Study Source groups, followed by a post-hoc analysis using Dunn's test with Bonferroni adjustment to identify which study sources differed significantly. Furthermore, we employed Pearson's chi-squared test to investigate potential pairwise associations between Accrual Success and Study Phase, and Fisher's exact test to assess possible association between Accrual Success and Study Source due to the lower number of observations (<5) in some categories. To examine associations with Accrual Success, we fitted all possible logistic regression models using different combinations of the three candidate predictors: Activation Days, Study Phase, and Study Source. The final model was selected based on the lowest Akaike Information Criterion (AIC) value. We also computed 95 % confidence intervals and odds ratios for the chosen model. All statistical analyses were conducted using specific packages in R (version 4.3.1), including *dplyr* [17], *ggpubr* [18], *gridExtra* [19], *janitor* [20], and *RColorBrewer* [21].

## 3 Results

After cleaning the data, 315 studies remained out of 720. Of these, 130 of 315 (42 %) were classified as successful when *k* = *0.5*, 116 of 315 (37 %) when *k* = *0.7* (37 %), and 79 of 315 (25 %) when *k* = *0.9*. Accordingly, 185 studies (58.7 %) failed to meet the accrual success goal at k = 0.5, 199 (63.2 %) at k = 0.7, and 236 (74.9 %) at *k* = *0.9*. Additionally, each study is categorized as either early-phase or late-phase, as described in Equation (3). Of these, 110 studies (34.9 %) were early-phase, and 205 (65.1 %) were late-phase. All subsequent analyses are based on this final cohort of 315 studies.

The minimum and maximum values for Activation Days recorded during the study period are 13 and 527 business days, respectively. The median number of Activation Days is 171, while the mean is 183.5, both of which are shorter than a business year, which typically consists of 252 days. The distribution of Activation Days is positively skewed (skewness = 0.88). The Shapiro-Wilk test for normality (S = 0.92, p-value <0.001) indicates that the distribution is not normal, with a few studies having exceptionally long activation times.

Fig. 1 illustrates the distribution of the 315 studies received by KUCC, categorized by study source and year. The number of studies increased steadily from 2018 to 2019. However, there was a sudden 37 % drop in 2020, followed by a 73 % decrease in 2021 and a 70 % decrease in 2022, likely due to the persistent effects of the COVID-19 pandemic. The majority of studies (199; 63 %) were sponsored by industrial or pharmaceutical sources, followed by 85 studies (27 %) from national, cooperative groups, or consortium sources. Only four studies (1.3 %) were funded through Externally Peer-Reviewed/Federal sources throughout the study period.

**Fig. 1:**
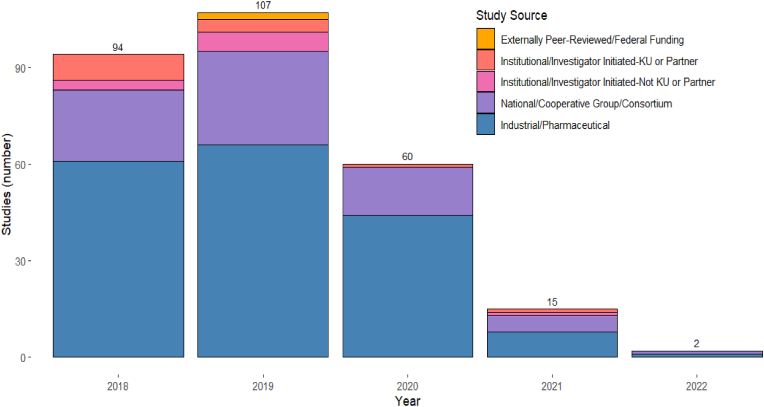
Trends in studies by study sources (2018–2022).

The median number of activation days varied notably across study sources. Institutional/Investigator Initiated: External (non-KU or Partner) studies had the highest median activation time, at 244 days, followed by studies funded through Externally Peer-Reviewed/Federal sources. Industrial/Pharmaceutical studies had a median activation time of 188 days, while Institutional/Investigator Initiated: Internal (KU or partner) studies showed a shorter median of 119 days. The lowest median activation time was observed in National/Cooperative Group/Consortium studies, at 89 days. Notably, Institutional/Investigator Initiated: Internal studies had a median activation time below the 120-day benchmark, and National/Cooperative Group/Consortium studies had a median below the 90-day mark. A Kruskal–Wallis test indicated a statistically significant difference in median activation days among the different study sources (H = 97.46, p < 0.001). Post-hoc comparisons using Dunn's test with Bonferroni adjustment revealed that studies funded by National/Cooperative Group/Consortium sources had significantly shorter Activation Days compared to those sponsored by Industrial/Pharmaceutical, Externally Peer-Reviewed/Federal Funding, and Institutional/Investigator Initiated: External (non-10.13039/100007859KU or Partner) sources.

In this study, we utilized three different threshold values (k={0.5,0.7,0.9}) to define the Accrual Success variable. The remainder of these results are based on the k=0.7 threshold value; the results for threshold values of k=0.5 and k=0.9 may be found in the Supplementary Materials. Studies with a shorter median activation time of 140.5 days are more likely to meet their accrual goals, while those with a longer median activation time of 187 days are less likely to meet their accrual targets. Based on the Wilcoxon rank-sum test results (W = 14,058, p-value <0.001), it can be concluded at a 0.05 significance level that the median Activation Days tend to be higher for the failed studies than for the successful studies. Furthermore, the results of the Wilcoxon rank-sum test conducted for the Study Phase (W = 13,607, p-value = 0.001) indicate, at the 0.05 level, that the median activation days for early-phase projects (182.5) are significantly greater than those for late-phase projects (166). In line with this finding, the Wilcoxon rank-sum test conducted for successful studies by study phase (W = 1,860, p = 0.002) indicates that the median activation days for early-phase successful studies (161.5) are significantly greater than those for late-phase successful studies (121.5). Together, these results suggest that the observed difference in activation times between early-phase and late-phase studies persists even when conditioning on study success. Fig. 2 illustrates that, across both early-phase and late-phase studies, successful studies consistently have lower median Activation Days compared to their unsuccessful counterparts.

**Fig. 2:**
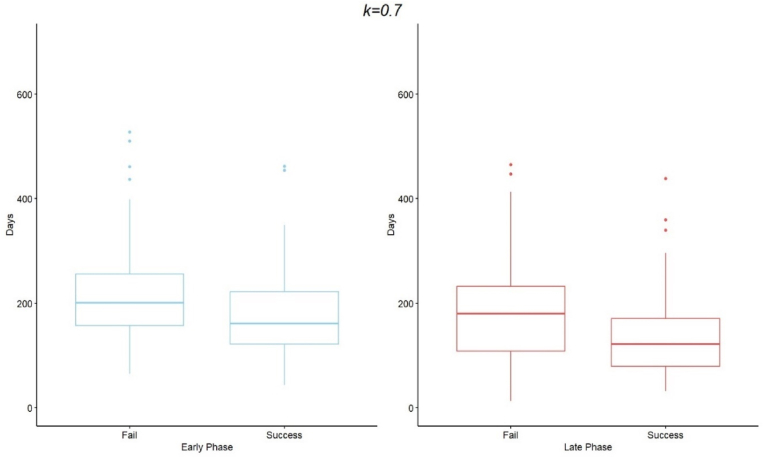
Box and whisker plots of activation days for accrual success by study phase.

Table 1 presents the Pearson's chi-squared test results for the pairwise association between Accrual Success and Study Phase, and Table 2 shows the Fisher's Exact test results for the association between Accrual Success and Study Source. According to Pearson's chi-squared test, it can be concluded with 95 % confidence that there is a significant association between Study Success and Study Phase. However, Fisher's Exact test suggests that there is no evidence to support a significant association between Study Source and Accrual Success with 95 % confidence.

**Table 1:** Chi-squared test results for association between accrual success and study phase.

Study Phase	Accrual Success	
	fail	success
early-phase	62 (56 %)	48 (44 %)
late-phase	147 (72 %)	58 (28 %)
$Pearson^{\prime }s\;\chi^{2}\;test\;p-value=0.0087$

**Table 2:** The Fisher's test results for association between accrual success and study source.

Study Source	Accrual Success	
	fail	success
Externally Peer-Reviewed/Federal Funding	2 (50 %)	2 (50 %)
Industrial/Pharmaceutical	137 (69 %)	62 (31 %)
Institutional/Investigator Initiated: Internal	7 (54 %)	6 (46 %)
Institutional/Investigator Initiated: External	12 (86 %)	2 (14 %)
National/Cooperative Group/Consortium	51 (60 %)	34 (40 %)
$Fisher^{\prime }s\;Exact\;test\;p-value=0.1801$		

The chi-squared test indicates a significant association between Accrual Success and Study Phase.

We fitted a logistic regression model to explore the linear relationship between the logit of the probability of Accrual Success (Y) and predictor variables, including Study Phase ($X_{1}$), Activation Days ($X_{2}$), and Study Source ($X_{3}$). All possible combinations of the three predictors were fitted, and based on the lowest AIC value of 385.58, the following model was selected.

(4) $$logit\left(P\left(Y=Yes|X_{1},X_{2}\right)\right)=0.842-0.902*X_{1}-0.005*X_{2}\text{.}$$

Here, the logit function transforms the probability of Accrual Success into a linear combination of predictor variables ($X_{1}$ and $X_{2}$) by taking the natural logarithm of the odds, where the odds represent the ratio of the probability of success to the probability of failure. Formally:

$$logit\left(P\left(Y=Yes|X_{1},X_{2}\right)\right)=\ln\left(\frac{P\left(Y=Yes|X_{1},X_{2}\right)}{1-P\left(Y=Yes|X_{1},X_{2}\right)}\right)\text{,}$$

where $X_{1}$ is the Study Phase, and $X_{2}$ is Activation Days. The Odds of Accrual Success $\frac{P\left(Y=Yes|X_{1},X_{2}\right)}{1-P\left(Y=Yes|X_{1},X_{2}\right)}$, quantifies how likely success is compared to failure for the specified values of $X_{1}$ and $X_{2}$.

According to the results in Table 3, it can be concluded, with 95 % confidence, that as the number of Activation Days increases, the odds of success for a study decrease by 0.5 % per day. Additionally, the odds of success for late-phase studies are approximately 41 % of those for early-phase projects. In other words, as the time to activate a study increases, the odds of project success decrease, and early-phase projects exhibit higher odds of success compared to late-stage projects.

**Table 3:** Logistic regression model results for accrual success.

Covariate		N	Odds Ratio	95 % Confidence Interval	p-value
Study Phase	early	110	*Reference category*		
	late	205	0.406	(0.242, 0.676)	<0.001
Activation Days		315	0.995	(0.991, 0.997)	<0.001

Early-phase was used as the reference group; both Study Phase and Activation Days are significant terms.

Fig. 3 illustrates the estimated probability of success and its variation with Activation Days and Study Phase. The estimated probability of success decreases with the number of Activation Days, with early-phase projects showing a higher probability than late-phase projects.

**Fig. 3:**
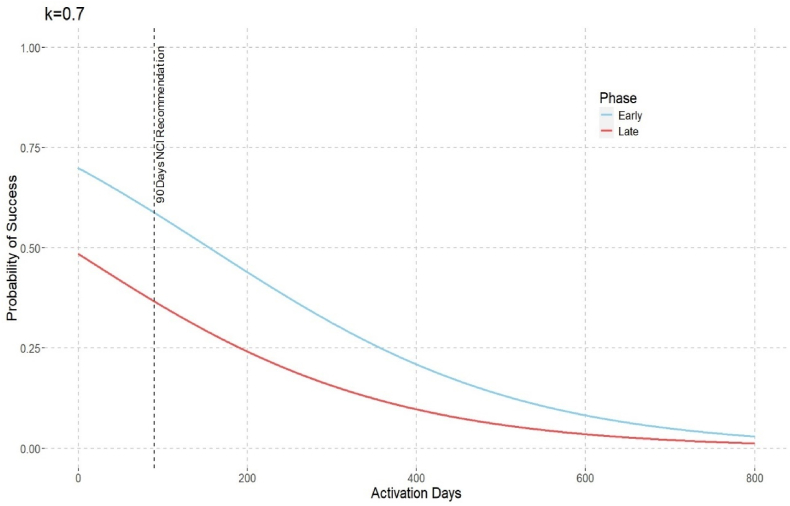
Estimated Probability of Success vs. Activation Days by Study Phase.

Based on NCI recommendations, the ideal study startup time is 90 days. Using the model equation (Equation (4)), we calculate that the estimated probability of success at 90 days of activation is notably higher for early-phase projects than for late-phase projects.

$$P\left(Y=Yes|X_{1}=late-phase,X_{2}=90\right)=0.38\text{,}$$

$$P\left(Y=Yes|X_{1}=early-phase,X_{2}=90\right)=0.60\text{.}$$

## 4 Discussion

Based on our analysis, studies with longer activation periods are less likely to achieve accrual success. Delays in activation often contribute to poor accrual outcomes and are commonly driven by factors such as complexity, prolonged timelines, excessive administrative burdens, conservative decision-making, and procedural inconsistencies [7,22,23]. To improve activation timelines and enhance accrual success, effective interventions include implementing parallel processes instead of sequential steps, regularly reviewing activation timelines, reducing unnecessary bureaucracy, and adopting automation tools and technologies [1,24].

Our study indicates that investigator-initiated external studies had the longest median activation times, while those initiated internally or through national/cooperative groups had the shortest. However, in our data, early-phase studies had longer median activation times than late-phase ones. Typically, late-phase trials require longer activation periods due to greater protocol complexity, multicenter coordination, and regulatory oversight involved in advanced-phase research [25]. In contrast, our findings show that early-phase projects exhibited longer activation times yet had higher odds of success compared to late-phase projects. This inverse pattern suggests that, when conditioning on study success, late-phase studies with relatively shorter activation times were more likely to be successful, thereby reversing the overall phase comparison and illustrating a form of Simpson's paradox [26].

Studies that clearly define their research goals, inclusion and exclusion criteria, scope, recruitment strategies, and consent documents often obtain IRB approvals within the expected timeframe. Study startup times can be streamlined by strategies such as enrolling participants at optimal points in the study design process and ensuring alignment with clearly defined goals and criteria from the start [9]. Based on our findings and existing literature, one effective strategy is the implementation of a rigorous, local institutional scientific review process. Scientific Review Committees (SRCs) that evaluate feasibility and scientific merit early in the process may help reduce downstream delays and ensure operational readiness before IRB submission [13]. While our study focused on the study phase and sponsor type as key factors influencing study startup, further research is needed to explore additional contributors that may delay the startup process.

Our analysis revealed a statistically significant association between Study Phase and Study Success, with early-phase trials (Phase I/II) demonstrating a higher likelihood of meeting accrual goals compared to late-phase studies (Phase III). Within KUCC's portfolio, this finding is consistent with internal observations where early-phase trials often receive higher prioritization scores due to their scientific novelty, innovation potential, and typically smaller, more targeted accrual requirements. In contrast, late-phase trials are often more complex, require broader eligibility criteria, and involve greater regulatory and logistical burdens, all of which may contribute to challenges in achieving accrual success. These local findings align with prior literature indicating that early-phase oncology trials tend to accrue more efficiently and are more responsive to institutional support strategies than later-phase trials, which face more external constraints and regulatory delays [27,28]. Therefore, KUCC's prioritization model, in which scientific importance (in part reflected in the study phase) influences resource allocation. These allocations appear to be a valid and evidence-informed approach to optimizing trial performance.

In addition to the study phase, our findings show a strong association between study source and activation timelines, however, not with accrual success. Specifically, investigator-initiated external studies had the longest median activation times, likely due to multi-institutional coordination, complex contractual negotiations, and limited administrative support at the initiating site. In contrast, studies initiated internally or through national cooperative groups demonstrated shorter activation times, likely reflecting streamlined internal processes, greater institutional familiarity with protocols, and more robust resourcing. While we observed descriptive differences in accrual outcomes by study source, these did not reach statistical significance, suggesting that activation delays may not directly translate to differences in accrual success. These findings underscore the importance of distinguishing operational factors that delay trial initiation from those that ultimately determine accrual outcomes.

Recognizing the challenges of clinical trial activation, the KU Cancer Center (KUCC) leadership team has adopted a strategic approach to reducing study start-up timelines by implementing a structured scoring algorithm. KUCC developed a study prioritization mechanism that evaluates protocols across six key domains: (1) scientific importance, reflected in the phase and novelty of the study—e.g., early-phase trials (Phase I/II) that explore novel mechanisms or therapies may be scored higher due to their potential to advance scientific discovery or fill critical unmet needs; (2) portfolio relevance, including whether the study addresses gaps in our current trial offerings; (3) significance, defined as the broader contribution to clinical science, patient care, or health equity; (4) funding source, whether externally sponsored, internally supported, or unfunded; (5) study type, such as federally funded or cooperative group trials; and (6) projected annual accrual. Based on the cumulative score, studies are categorized as high (>50), medium (30–50), or low priority (<30). Higher-scoring studies are prioritized during the start-up process, allowing the Clinical Trials Office to allocate resources toward trials with the greatest anticipated impact and strategic value. Continued innovation in prioritization methods like this helps to streamline activation timelines and improve trial efficiency [1]. Importantly, minimizing startup delays requires a coordinated, team-based effort across all stakeholders involved in protocol review, regulatory, and operational workflows.

## 5 Limitations

This study period (2018–2022) overlaps with the COVID-19 pandemic and the mid-2020 implementation of the TRAX system, both of which may have influenced activation timelines. As TRAX was not consistently applied across the full study window, its effect on activation could not be fully evaluated, potentially limiting generalizability. However, because our primary aim was to assess associations between study characteristics, activation times, and their negative impact on Accrual, we believe the observed patterns remain informative. As a future study, we will evaluate the impact of TRAX implementation through a pre- and post-intervention analysis.

## 6 Conclusion

Study startup is a crucial step for every clinical trial, and developing a robust process to streamline regulatory, contracting, and financial negotiations is essential for achieving quicker study activation. The initial study startup time can often disrupt the momentum of the study team, resulting in delays. Our study found that trials with shorter activation times were significantly more likely to achieve their accrual goals, whereas those with longer activation times had a higher likelihood of failing to meet accrual targets. It has been concluded that there is a significant association between Study Success and Study Phase. To identify the delays in study startup, cancer centers nationwide should establish process repositories that set clear expectations for sponsors and investigators regarding timelines. Implementing a study tracker dashboard, similar to KUCC, can enhance transparency and help identify and address bottlenecks. It may also be necessary for Cancer Center leadership to make strategic decisions about pursuing studies based on their potential for quick activation. Furthermore, introducing scoring metrics for study intake to prioritize study activation can help manage workload and staffing challenges. By addressing barriers that impede the approval process, we can facilitate quicker approvals, ultimately increasing the success of studies regardless of their phase.

## CRediT authorship contribution statement

**Isuru Ratnayake:** Writing – review & editing, Writing – original draft, Supervision, Methodology. **Anh-Tuan Do:** Visualization, Formal analysis. **Daniel Gajewski:** Visualization, Formal analysis. **Sam Pepper:** Writing – original draft, Software, Formal analysis, Data curation. **Oluwatobiloba Ige:** Formal analysis, Data curation. **Natalie Streeter:** Writing – review & editing, Supervision. **Tara L. Lin:** Writing – review & editing, Supervision. **Matthew McGuirk:** Writing – original draft, Formal analysis, Data curation. **Byron Gajewski:** Writing – review & editing, Supervision. **Dinesh Pal Mudaranthakam:** Writing – review & editing, Writing – original draft, Supervision, Software, Resources, Conceptualization.

## Ethics approval and consent to participate

Not applicable.

## Declaration of Generative AI and AI-assisted technologies in the writing process

During the preparation of this work, the author(s) used ChatGPT to improve the language and readability. After using this tool, the author(s) reviewed and edited the content as needed and take(s) full responsibility for the content of the publication.

## Funding

10.13039/100000054National Cancer Institute 10.13039/100007345Cancer Center Support Grant P30CA168524 supported this study.

## Declaration of competing interest

The authors declare that they have no known competing financial interests or personal relationships that could have appeared to influence the work reported in this paper.

## Figures and Tables

**figs1:**
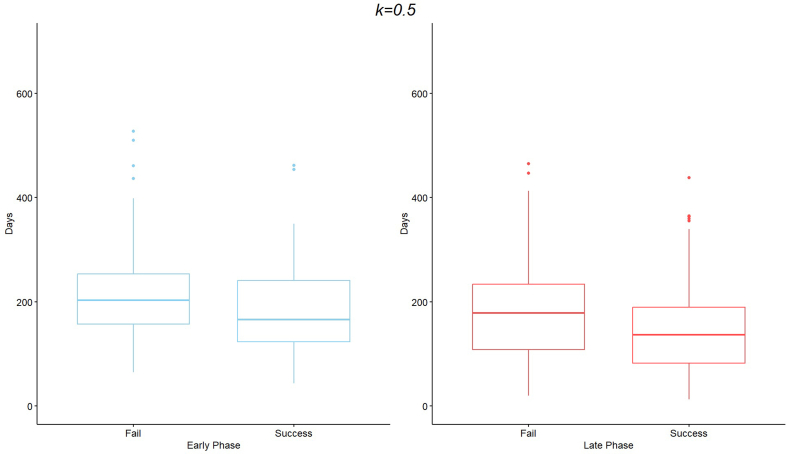


**figs2:**
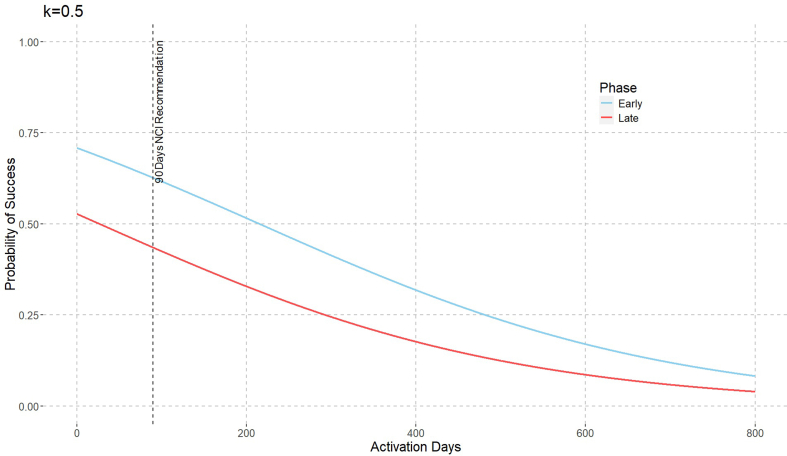


**figs3:**
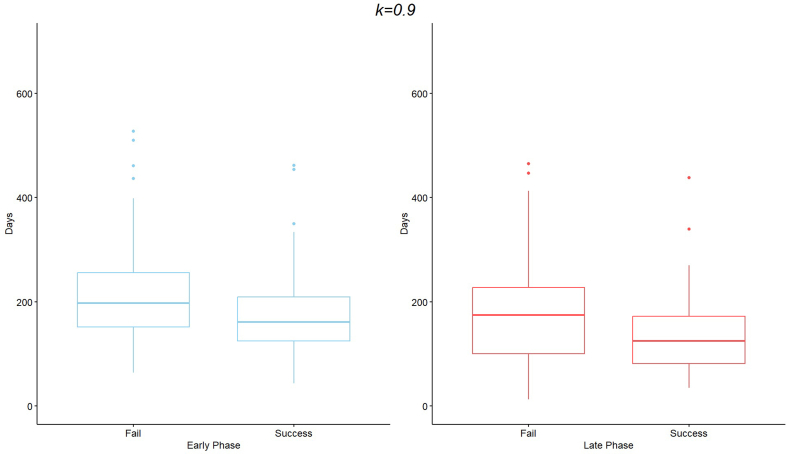


**figs4:**
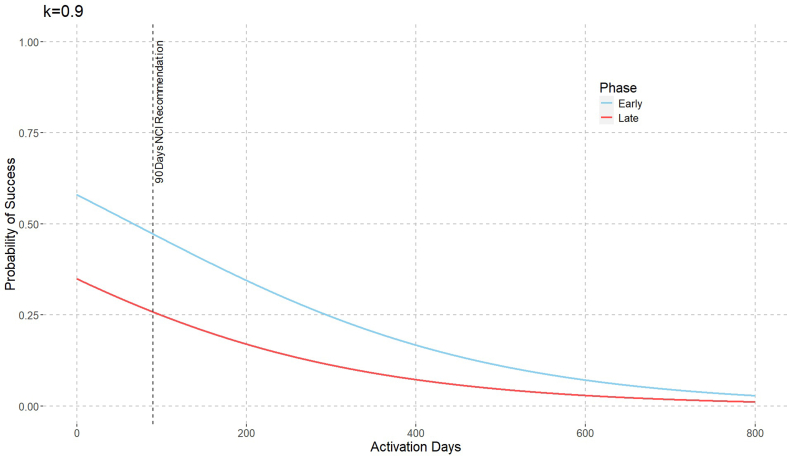


## Data Availability

Data will be made available on request.

## List of abbreviations:

***AACI***: Association of American Cancer Institutes
***AIC***: Akaike Information Criteria
***CTMS***: Clinical Trial Data Management System
***DWG***: Disease Working Group
***ERC***: Executive Resourcing Committee
***IRB***: Institutional Review Board
***KUCC***: The University of Kansas Cancer Center
***NCI***: National Cancer Institute
***PRMC***: Protocol Review and Monitoring Committee
***SRC***: Scientific Review Committee
***TRAX***: Trial Review and Approval for Execution
