# Supplementary material for: Evaluating the impact of delayed study startup on accrual in cancer studies
Source: Contemp Clin Trials Commun. 2025 Oct 23;48:101562. doi: 10.1016/j.conctc.2025.101562 (PMC12595353; doi:10.1016/j.conctc.2025.101562)
Supplement: Multimedia component 1 [file mmc1.docx]

**Supplementary Materials**

**Accrual Success at** $\boldsymbol{k=0.5}$

The results in Table S1 show a significant association between Accrual Success and Study Phase, as confirmed by Pearson's chi-squared test with 95% confidence. However, Table S2 reveals no significant association between Accrual Success and Study Source, as indicated by the Fisher's exact test at a 95% confidence level. In Table S3, the results indicate with a 95% confidence level that an increase of one activation day is associated with a 0.4% decrease in the odds of accrual success. Moreover, late-phase studies exhibit approximately 46% odds of success compared to that of the early-phase projects. In summary, longer activation times are linked to reduced project success, and early-phase studies tend to have higher odds of success compared to late-phase projects.

**Table S1:** Chi-Square Test Results for Association Between Accrual Success and Study Phase when $k=0.5$.

| **Study Phase** | **Accrual Success** | |
| --- | --- | --- |
|  | fail | success |
| early-phase | 54 (49%) | 56 (51%) |
| late-phase | 131 (64%) | 75 (36%) |
| $Pearson^{'}s \chi^{2} p-value=0.0153$ | | |

The chi-squared test indicates significant association between Accrual Success and Study Phase.

**Table S2:** The Fisher’s Test Results for Association Between Accrual Success and Study Source when $k=0.5$.

| **Study Source** | **Accrual Success** | |
| --- | --- | --- |
|  | fail | success |
| Externally Peer-Reviewed/Federal Funding | 1 (25%) | 3 (75%) |
| Industrial/Pharmaceutical | 123 (62%) | 76 (38%) |
| Institutional/Investigator Initiated: Internal | 6 (46%) | 7 (54%) |
| Institutional/Investigator Initiated: External | 11 (79%) | 3 (21%) |
| National/Cooperative Group/Consortium | 44 (52%) | 41 (48%) |
| $Fisher^{'}s Exact test p-value=0.1065$ | | |

The Fisher's test shows no significant association between Accrual Success and Study Source.

**Table S3:** Logistic Regression Model of Predictors of Accrual Success when $k=0.5$.

| Covariate | | N | Odds Ratio | 95% Confidence Interval | p-value |
| --- | --- | --- | --- | --- | --- |
| Study Phase | early | 110 | *Reference* category | | |
|  | late | 205 | 0.459 | (0.279, 0.749) | <0.001 |
| Activation Days | | 315 | 0.996 | (0.993, 0.998) | <0.001 |

Early-phase was used as the reference group, both Study Phase and Activation Days are significant terms.

When $k=0.5$, a study successfully reaches its accrual goal, it typically takes a median of 149.5 Activation Days. In contrast, studies that fail to meet their accrual goal experience a longer median of 184 Activation Days. Figure S1 illustrates that, across both early-phase and late-phase studies, successful studies consistently have lower median Activation Days compared to their unsuccessful counterparts.

**Figure S1:** Box and Whisker Plots of Activation Days for Accrual Success by Study Phase when $k=0.5$.

Figure S2 displays the relationship between success probability, activation days, and study phase. It is evident from the figure that as the activation days increase, the probability of success decreases. Furthermore, early-phase projects demonstrate a significantly higher probability of success compared to their late-phase counterparts.

**Figure S2:** Probability of Success vs. Activation Days by Study Phase when $k=0.5$.

**Accrual Success at** $\boldsymbol{k=0.9}$

In Table S4, a significant association between Accrual Success and Study Phase is evident, confirmed by Pearson's chi-squared test with 95% confidence. However, Table S5 does not provide strong evidence of an association between Accrual Success and Study Source, as indicated by the Fisher's exact test at a 95% confidence level. Table S6, the results indicate that, with 95% confidence, an increase of one activation day is associated with a 0.5% decrease in the odds of accrual success. Additionally, late-phase studies exhibit approximately 39% odds of success compared to that of the early-phase projects. In summary, extended activation times are linked to reduced project success, while early-phase studies tend to have higher odds of success compared to late-phase projects.

**Table S4:** Chi-Square Test Results for Association Between Accrual Success and Study Phase when $k=0.9$.

| **Study Phase** | **Accrual Success** | |
| --- | --- | --- |
|  | fail | success |
| early-phase | 72 (65%) | 38 (35%) |
| late-phase | 164 (80%) | 41 (20%) |
| $Pearson^{'}s \chi^{2} test p-value=0.0069$ | | |

The chi-squared test indicates significant association between Accrual Success and Study Phase.

**Table S5:** The Fisher’s Test Results for Association Between Accrual Success and Study Source when $k=0.9$.

| **Study Source** | **Accrual Success** | |
| --- | --- | --- |
|  | fail | success |
| Externally Peer-Reviewed/Federal Funding | 2 (50%) | 2 (50%) |
| Industrial/Pharmaceutical | 153 (77%) | 46 (23%) |
| Institutional/Investigator Initiated: Internal | 9 (69%) | 4 (31%) |
| Institutional/Investigator Initiated: External | 13 (93%) | 1 (7%) |
| National/Cooperative Group/Consortium | 59 (69%) | 26 (31%) |
| $Fisher^{'}s Exact test p-value=0.1845$ | | |

The Fisher's test shows no significant association between Accrual Success and Study Source.

**Table S6:** Logistic Regression Model of Predictors of Accrual Success when $k=0.9$.

| Covariate | | N | Odds Ratio | 95% Confidence Interval | p-value |
| --- | --- | --- | --- | --- | --- |
| Study Phase | early | 110 | *Reference category* | | |
|  | late | 205 | 0.389 | (0.224, 0.671) | <0.001 |
| Activation Days | | 315 | 0.995 | (0.992, 0.998) | <0.001 |

Early-phase was used as the reference group, both Study Phase and Activation Days are significant terms.

When $k=0.9$, a study successfully reaches its accrual goal, it typically has a median of 140 Activation Days. Conversely, studies that fail to meet their accrual goal experience a longer median of 182.5 Activation Days. Figure S3 depicts that, spanning both early-phase and late-phase studies, successful studies consistently exhibit lower median Activation Days compared to their unsuccessful counterparts.

**Figure S3:** Box and Whisker Plots of Activation Days for Accrual Success by Study Phase when $k=0.9$.

Figure S4 displays the relationship between success probability, activation days, and study phase. It is evident from the figure that as the activation days increase, the probability of success decreases. Furthermore, early-phase projects demonstrate a significantly higher probability of success compared to their late-phase counterparts.

**Figure S4:** Probability of Success vs. Activation Days by Study Phase when $k=0.9$.
